# Long-Term Diet Supplementation with *Lactobacillus paracasei* K71 Prevents Age-Related Cognitive Decline in Senescence-Accelerated Mouse Prone 8

**DOI:** 10.3390/nu10060762

**Published:** 2018-06-13

**Authors:** Henry M. Corpuz, Saki Ichikawa, Misa Arimura, Toshihiro Mihara, Takehisa Kumagai, Takakazu Mitani, Soichiro Nakamura, Shigeru Katayama

**Affiliations:** 1 Interdisciplinary Graduate School of Science and Technology, Shinshu University, 8304 Minamiminowa, Kamiina, Nagano 399-4598, Japan; 15st553b@shinshu-u.ac.jp (H.M.C.); snakamu@shinshu-u.ac.jp (S.N.); 2 Rice Chemistry and Food Science Division, Philippine Rice Research Institute, Maligaya, Science City of Muñoz, Nueva Ecija 3119, Philippines; 3 Faculty of Agriculture, Shinshu University, 8304 Minamiminowa, Kamiina, Nagano 399-4598, Japan; 18as202a@shinshu-u.ac.jp (S.I.); 17as202g@shinshu-u.ac.jp (M.A.); 4 Kameda Seika Co., Ltd., 3-1-1 Kameda-Kogyodanchi, Konan-ku, Niigata-shi, Niigata 950-0198, Japan; t_mihara@sk.kameda.co.jp (T.M.); t_kumagai@sk.kameda.co.jp (T.K.); 5 Interdisciplinary Cluster for Cutting Edge Research, Shinshu University, 8304 Minamiminowa, Kamiina, Nagano 399-4598, Japan; mitani@shinshu-u.ac.jp

**Keywords:** probiotics, *Lactobacillusparacasei*, cognitive dysfunction, brain-derived neurotrophic factor, serotonin

## Abstract

This study aimed to assess the suppressive effect of long-term diet supplementation with *Lactobacillus* strains on cognitive decline in the senescence-accelerated mouse prone 8 (SAMP8) model. For 43 weeks, fourteen-week-old female SAMP8 mice were fed a standard diet containing 0.05% (*w*/*w*) *Lactobacillus casei* subsp. *casei* 327 (L. 327) or *Lactobacillus* *paracasei* K71 (L. K71) derived from rice grains and sake lees, respectively. SAMP8 mice that were fed a L. K71-supplemented diet had better cognitive performance compared with the control and L. 327 groups in the Barnes maze and passive avoidance tests. An ELISA analysis revealed that the levels of serotonin were elevated in the serum and brain tissue of L. K71-fed mice. The protein expression levels of brain-derived neurotrophic factor (BDNF), cAMP response element binding protein (CREB), and phosphorylated CREB were evaluated using western blot. Long-term administration of L. K71 resulted in increased protein expression of BDNF and CREB phosphorylation in the hippocampus. These results suggest that prolonged intake of a diet supplemented with a *Lactobacillus* strain derived from sake lees may prevent age-dependent cognitive decline by upregulating BDNF expression in the hippocampus.

## 1. Introduction

Probiotics are defined as living microorganisms that confer health benefits on the host when administered in adequate amounts. The intestinal microbiota converts dietary nutrients into biologically active metabolites affecting regulatory functions in the host. Probiotics help restore gut microbial diversity and its host-beneficial functions, resulting in amelioration or prevention of gut inflammation [1,2] and other intestinal or systemic disease phenotypes [3,4].

Recently, interest has been growing in the potential beneficial effects of dietary probiotics on behavior, mood, and mental health. In particular, with an increasingly aging population, the risk of illness or death caused by cognitive decline among older people has been increasing. Certain *Lactobacillus* and *Bifidobacterium* have been shown to improve gut health, as well as alleviate mood disorders and stress-induced behavioral changes. Several studies have shown that ingestion of some probiotics can not only rescue stress-related disorders but can also improve cognitive performance. Several *Bifidobacterial* strains (e.g., *B. longum* 1714, *B. breve* 1205) can induce positive effects on cognition in fear-related cognitive tasks by decreasing anxiety in mice [5]. Administration of *Lactobacillus helveticus* NS8 in rats also ameliorated behavioral (anxiety and depression) and cognitive dysfunction induced by chronic restraint stress [6]. Distrutti et al. [7] showed that the age-related deficit in long-term potentiation was markedly attenuated in rats that received a mixture of eight different strains of bacteria. These findings suggest that daily intake of probiotics can improve cognitive functions.

Sake lees are byproducts of Japanese rice wine production; large quantities of this residue are discarded as industrial waste. Sake lees are also known as a viable source of beneficial microorganisms, including lactic acid bacteria. Recently, *Lactobacillus paracasei* K71 (L. K71) has been isolated from sake lees, and Saito et al. [8] reported that L. K71 has an immunomodulatory potential. Intake of a dietary supplement containing heat-killed L. K71 has been reported to reduce the clinical severity of atopic dermatitis in a randomized controlled trial and enhance secretory immunoglobulin A release in the saliva. *Lactobacillus casei* subsp. *casei* 327 (L. 327) is another lactic acid bacterium discovered in rice grain. Consumption of heat-killed L. 327 was effective in improving skin conditions of healthy female volunteers [9].

The senescence-accelerated mouse (SAM) is an accelerated aging model established through phenotypic selection from a common genetic pool of the AKR/J strain [10]. The unique characteristic of SAM prone 8 (SAMP8) mice is a low incidence of phenotypic changes accompanying age-related cognitive impairment [11]. Therefore, SAMP8 has been widely accepted as a good animal model to investigate the effects of environmental factors, such as food intake and exercise on age-related learning and memory deficits. In the present study, we investigated whether long-term diet supplementation with *Lactobacillus* strains isolated from rice or sake lees could attenuate spatial learning deficits and memory loss in aged SAMP8 mice.

## 2. Materials and Methods

### 2.1. Animals

Fourteen-week-old female SAMP8 mice were purchased from Japan SLC, Inc. (Shizuoka, Japan). All mice were housed in groups of four per cage and the animal room was maintained at a controlled temperature (20−23 °C), humidity (40−70%), and with an alternating 12 h/12 h light-dark cycle (lights on at 8:00 a.m.). All experiments were performed in accordance with the animal experiment protocol approved by the Institutional Animal Care and Use Committee of Shinshu University (Permit No. 270076). 

### 2.2. Animal Protocol

Mice were divided into 3 groups: Control (*n* = 12), *Lactobacillus casei* subsp. *casei* 327-fed group (L. 327; *n* = 12), and *Lactobacillus paracasei* K71-fed group (L. K71; *n* = 12). The control group was fed an AIN-93M diet (Oriental Yeast, Tokyo, Japan) only, and the L. 327 and L. K71 groups were fed an AIN-93M diet containing 0.05% (*w*/*w*) of the respective heat-killed *Lactobacillus* strain. The mice were allowed free access to food and tap water. Food intake and body weight were recorded every week. At 54- to 57-weeks-old, all mice were subjected to the Barnes maze, passive avoidance, and Y-maze tests to assess their cognitive performance. Mouse feces were collected and all mice were sacrificed by an overdose of isoflurane 3–5 days after the last memory test, and the blood, hippocampus, and cerebral cortex were collected, frozen in liquid nitrogen, and kept at −80 °C until analysis.

### 2.3. Barnes Maze Test

**Figure 1:**
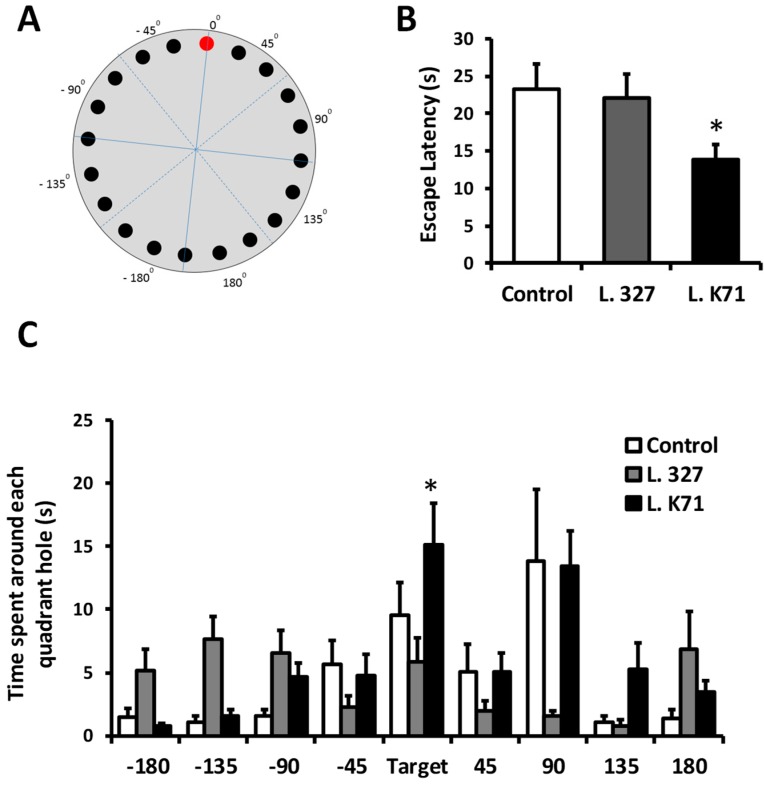
Effect of 43-week *Lactobacillus* strain administration on spatial learning and memory in senescence-accelerated prone 8 mice (SAMP8). (**A**) Barnes maze diagram used to interpret the data; (**B**) Escape latency during the probe test; (**C**) Time spent in each quadrant during the probe test. Data are presented as mean ± SEM; *n* = 10 mice per group; * *p* < 0.05 vs. the control group.

The Barnes maze test was performed to assess spatial learning in SAMP8 mice. The maze consisted of a gray platform (90 cm in diameter) with 20 holes (5 cm in diameter) located 3 cm from the perimeter (Muromachi Kikai Co. Ltd., Tokyo, Japan). A black escape box (EB) was placed under one of the holes. This circular platform was mounted on top of a steel stool, 90 cm above the ground, and balanced. Visual cues were placed on the walls (triangle and square signs) of the experimental room. The maze was divided into 4 quadrants (45°, 90°, 135° and 180°) in clockwise and counterclockwise directions from the EB (0°) position (Figure 1A). The animals interacted with the Barnes maze in 3 phases: Habituation (1 day), training (4 days), and probe (1 day). 

Habituation: Mice were allowed to move freely on the platform, placed in the center of the maze, and guided to the EB, where they remained for 2 min to familiarize themselves with the maze and hidden box.

Training: All mice received 3 trials per day for 4 days. During training sessions, the mouse was placed in the middle of the maze under a box chamber for 10 s and then allowed to freely explore the platform until either it entered the EB or 5 min had elapsed. Mice were allowed to stay in the escape box for 1 min before being returned to their cages after each trial, with an inter-trial interval of 30 min. If the mouse did not enter the EB, it was returned to the maze center, gently guided to the EB, and allowed to stay in it for 1 min. Guiding mice into the EB is important to show them that it exists.

Probe test: On the probe day, the EB was removed from the maze, and mice were placed in the center of the maze under a black chamber for 10 s. Each mouse was given 2 min to explore the maze and search for the EB. The mouse was returned to its holding cage immediately after the test. During the probe test, escape latency (time to enter the EB) and stay-in-the-hole time in each quadrant were recorded.

### 2.4. Passive Avoidance Test

The passive avoidance test was conducted using a step-through test cage (Muromachi Kikai Co. Ltd.) consisting of white and black compartments separated by a sliding door. In the training phase, each mouse was placed in the light compartment and allowed to explore for 10 s. The door was opened, and the step-through latency was recorded. After the mice entered the dark compartment, the door was immediately closed and a mild foot shock of 0.2 mA was applied for 3 s. Training sessions were conducted for 2 consecutive days. The probe test was performed using the same procedure without any shock. The step-through latency to enter the dark compartment was recorded. A maximum retention latency of 300 s was allowed for mice that did not enter the dark compartment.

### 2.5. Y-Maze Test

The Y-maze was a 3-arm maze with equal angles between all arms (Muromachi Kikai Co. Ltd.). Mice were individually placed at the center of the maze and allowed to move freely through the maze for 5 min. Spontaneous alternations (defined as consecutive entries into all 3 arms without repetitions, in overlapping triplet sets) were recorded. The total number of arm entries was collected during the test period. The alternation percentage was calculated as the ratio of actual to possible alternations (defined as the total number of arm entries − 2) × 100.

### 2.6. ELISA Measurement of Serotonin Levels 

Blood samples were centrifuged at 1000× *g* for 15 min at room temperature, and the serum was collected and stored at −80 °C until analysis. A portion of the brain sample was weighed and homogenized in a stabilization buffer (0.05 N HCl with 0.1% ascorbic acid; 1:10, *w*/*v*). The homogenate was centrifuged at 14,000× *g* for 20 min at 4 °C, and the supernatant was passed through a 0.45 µm centrifugal filter (Ultra-free-MC-HV, Merck Millipore, Darmstadt, Germany) and Amicon Ultra 0.5 mL centrifugal filter unit (Ultracel-10K, Merck Millipore). Serotonin levels were measured in serum and brain supernatants using a Serotonin ELISA kit (ADI-900-175, Enzo Life Sciences, Farmingdale, NY, USA) according to the manufacturer’s instructions.

### 2.7. Gene Expression Analysis by Quantitative PCR (qPCR)

Total RNA and protein were isolated using Invitrogen TRizol reagent (Thermo Fisher Scientific, Inc., Waltham, MA, USA) according to the manufacturer’s instructions. The RNA samples were converted to double-stranded cDNA using ReverTra Ace (Toyobo, Osaka, Japan). Quantification was performed with a Kapa SYBR Fast qPCR kit (Kapa Biosystems, Woburn, MA, USA) and a TP850 Thermal Cycler Dice Real time system (Takara, Shiga, Japan). The forward and reverse primers for each gene of interest are summarized in Table 1. Fold changes in the relative mRNA expression level for each gene were calculated using the 2^−∆∆*C*t^ method, and the values were normalized to that of a housekeeping gene (*Actb*).

### 2.8. Western Blotting Analysis

The concentrations of proteins extracted with TRizol reagent (Thermo Fisher Scientific, Inc.) were determined using Bradford’s method with bovine serum albumin as a standard. Samples containing equal protein amounts and prestained molecular weight markers were separated by Tris-SDS-PAGE and transferred onto polyvinylidene fluoride membranes (0.45 µm, Merck Millipore). The membranes were blocked with 3% BSA in Tris-buffered saline with 0.05% Tween-20 for 1 h at room temperature, and incubated overnight at 4 °C with the following antibodies: rabbit polyclonal anti-brain-derived neurotrophic factor (BDNF; 1:3000; Abcam, Cambridge, MA, USA), rabbit monoclonal anti-cAMP response element binding protein (CREB; 1:2000; Abcam), rabbit monoclonal anti-Ser133-phosphorylated CREB (pCREB; 1:2000; Abcam), and mouse monoclonal anti-β-actin (1:5000; Santa Cruz Biotechnology, Dallas, TX, USA). Subsequently, the membranes were washed and incubated for 1 h at room temperature with secondary HRP-conjugated anti-rabbit (1:5000; Santa Cruz Biotechnology) or anti-mouse antibodies (1:10,000; Santa Cruz Biotechnology). Chemiluminescence detection was performed using the EzWestLumi plus kit (ATTO, Tokyo, Japan) and AE-9300 Ez-Capture (ATTO). Densitometric analyses were performed using the public domain NIH Image Program, ImageJ.

### 2.9. Immunostaining

Paraffin-embedded mouse brain sections were dewaxed using xylene and hydrated in ethanol at decreasing concentration. The sections were boiled in 10 mM Tris/1 mM EDTA buffer (pH 9.0) for 20 min and cooled down for 30 min at room temperature for antigen retrieval. The sections were washed two times with TBS solution. After one hour blocking with 5% BSA in TBS, the sections were incubated with antibody against BDNF (1:200, Abcam) overnight at 4 °C. The slides were washed two times in TBS and incubated with the secondary antibody, Alexa Fluor 488 goat anti-rabbit IgG (H&L) (1:100, Abcam). After washing two times with TBS solution, the sections were mounted with immunoselect antifading mounting medium DAPI (Dianova, Hamburg, Germany) and examined under fluorescence microscope (EVOS fl; Advanced Microscopy Group, Bothell, WA, USA).

### 2.10. Statistical Analysis

The GraphPad Prism 5.0 software (GraphPad software, San Diego, CA, USA) was used to perform statistical analyses. Data are represented as the means ± SEMs. Differences between the means were evaluated using ANOVA followed by the Bonferroni post hoc test for mean comparisons.

## 3. Results

### 3.1. Effect of Long-Term Administration of Lactobacillus Strains on Spatial Learning and Memory in SAMP8 Mice

**Figure 2:**
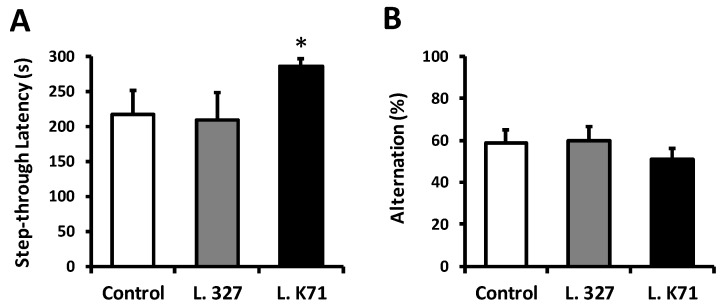
Effects of 43-week *Lactobacillus* strain administration on fear-motivated learning and short-term memory in SAMP8 mice. (**A**) Step-through latency in the passive avoidance test; (**B**) Spontaneous alternation behavior in the Y-maze test. Data are presented as mean ± SEM; *n* = 10 mice per group; * *p* < 0.05 vs. the control group.

The cognitive performance of SAMP8 mice was assessed after 43 weeks of feeding with diets supplemented with *Lactobacillus* strains isolated from rice and sake lees. Spatial learning and memory were evaluated using the Barnes maze test, performance in which is dependent on hippocampal functions. In this test, mice were trained to locate an EB hidden in one of the 20 holes located around the perimeter of an open circular platform. To find the EB, mice must learn, memorize, and use the relationships among the visual cues in the room. After 4 days of training sessions, L. K71 mice exhibited significantly shorter escape latency in searching for the EB during the probe test compared to the control and L. 327-fed group (Figure 1B). Moreover, the L. K71-fed group spent more time in the 0° hole, where the EB was located during the training phase (Figure 1C). A test for fear-motivated passive avoidance was also employed to evaluate associative memory in aged mice. In this task, memory performance is associated with the latency to enter a dark compartment where the mouse has been exposed to an electric shock. Therefore, the greater the latency, the better the memory retention. The step-through latency of the L. K71 group was significantly higher than those of the control and L. 327 groups (Figure 2A). Further, the short-term working memory of SAMP8 mice was evaluated with the Y-maze test. However, no significant differences were found in spontaneous alternation behavior among the mouse groups (Figure 2B). These results suggest that continuous and prolonged administration of the L. K71 strain, derived from sake lees, can attenuate cognitive decline in SAMP8 mice. 

### 3.2. Effect of Lactobacillus Strain Supplementation on Serotonin Levels in the Blood Serum and Brain of SAMP8 Mice

**Figure 3:**
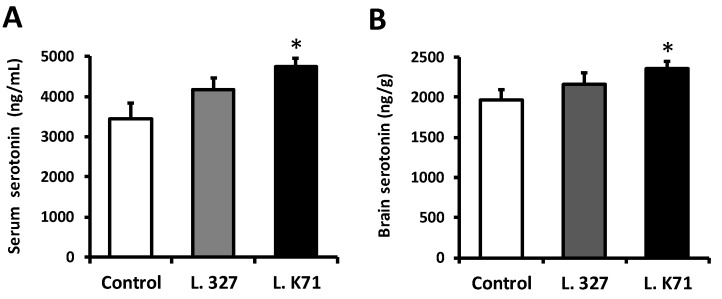
Effect of prolonged diet supplementation with *Lactobacillus* strains on the serotonin levels in the blood serum (**A**) and brain (**B**) of SAMP8 mice fed for 43 weeks diets containing *Lactobacillus* strains. Serotonin concentrations were measured by ELISA. Data are expressed as mean ± SEM; *n* = 8 mice per group; * *p* < 0.05 vs. the control group.

We next investigated the effect of long-term administration of a *Lactobacillus*-supplemented diet on the systemic and brain serotonin levels of SAMP8 mice. An ELISA analysis revealed that the serum serotonin level of L. K71-fed mice was significantly higher than that of the control and L. 327 groups (Figure 3A). A significant rise in the serotonin level was also observed in the brain extract of SAMP8 mice fed L. K71 (Figure 3B). While serotonin levels also increased in L. 327-fed mice, the levels of serotonin in both the blood serum and brain were not significantly different from those of the control group. We further investigated whether the observed differences in serotonin levels might be related to differences in food intake; however, the food intake and body weight dynamics did not differ among the groups throughout the feeding experiment (Figure S1). 

### 3.3. Effect of Prolonged Lactobacillus Supplementation on Serotonin Biosynthesis in the Hippocampus and Cortex

**Figure 4:**
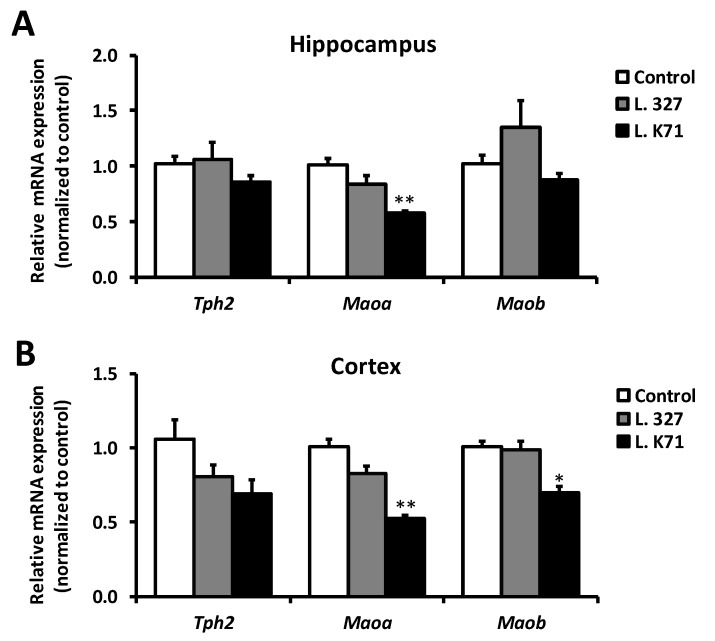
Expression of serotonin synthesis and degradation enzymes in the hippocampus (**A**) and cortex (**B**) of SAMP8 fed for 43 weeks diets containing *Lactobacillus* strains. Data are expressed as mean ± SEM; *n* = 8 mice per group; * *p* < 0.05, ** *p* < 0.01 vs. the control group. *Tph2*, tryptophan hydroxylase 2; *Maoa*, monoamine oxygenase A; *Maob*, monoamine oxygenase B.

We next assessed the effect of increased serotonin levels in the brain on serotonin synthesis and degradation enzymes. The mRNA expression level of tryptophan hydroxylase 2 (TPH2), the rate limiting enzyme in serotonin biosynthesis, did not differ among the treatments. In contrast, the mRNA expression level of monoamine oxygenase A (MAOA), responsible for serotonin degradation, was significantly downregulated in the hippocampus of the L. K71 group (Figure 4A). The mRNA expression of the other monoamine oxygenase isoform (MAOB) was slightly reduced in the L. K71 group, whereas no reduction in the *Maoa* and *Maob* mRNA levels was observed in the L. 327 group. In addition, a similar decrease in the *Maoa* mRNA level was also found in the cortex of SAMP8 mice after long-term supplementation with *Lactobacillus* strains (Figure 4B).

### 3.4. Effect of Lactobacillus Strain Supplementation on BDNF Expression in the Hippocampus and Cortex

**Figure 5:**
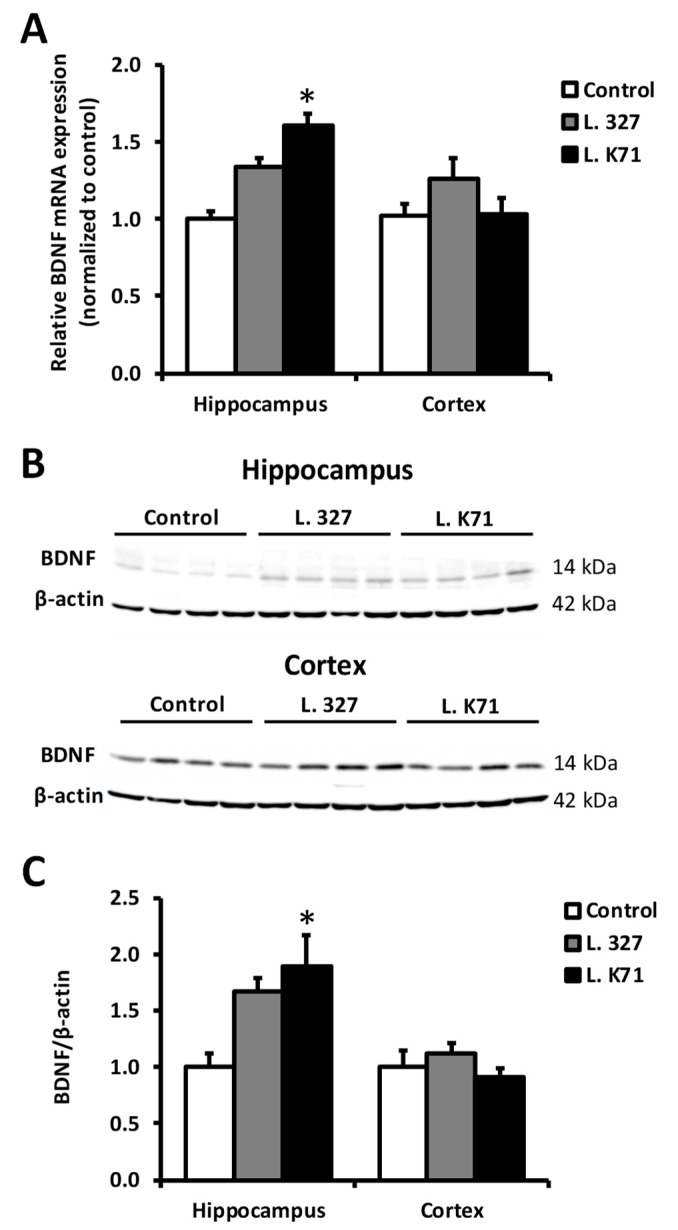
Effects of 43-week *Lactobacillus* strain administration on the expression levels of brain-derived neurotrophic factor (BDNF) in the hippocampus and cortex of SAMP8. (**A**) *Bdnf* mRNA levels measured by quantitative PCR (*n* = 8 mice per group); (**B**) Western blotting analysis (*n* = 4 mice per group) of the protein levels of BDNF; (**C**) Quantification of band intensities in (**B**). Data are expressed as mean ± SEM, * *p* < 0.05 vs. the control group.

**Figure 6:**
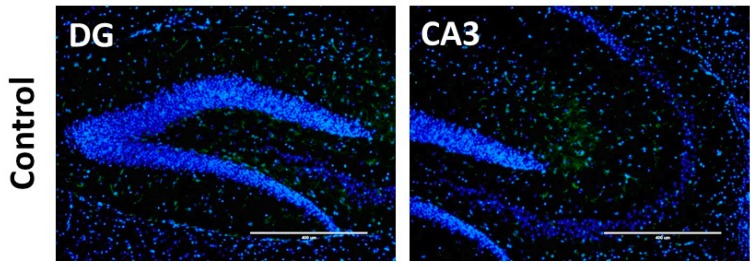

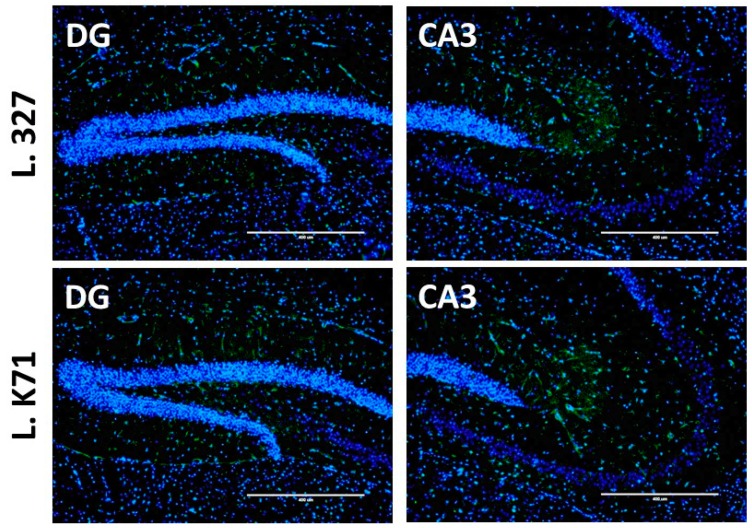
Representative images of BDNF immunofluorescence (green) in the hippocampal dentate gyrus (DG) and CA3 regions of SAMP8. DAPI stained nuclei (blue). Scale bar: 400 μm.

**Figure 7:**
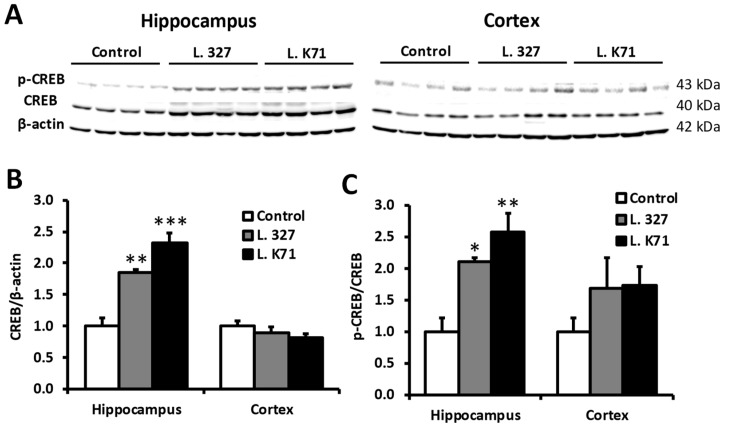
Effects of 43-week *Lactobacillus* strain administration on the expression levels of cAMP response element binding protein (CREB), and on CREB phosphorylation in the hippocampus and cortex of SAMP8. (**A**) Western blotting analysis (*n* = 4 mice per group) of the protein levels of CREB, and phosphorylated CREB (pCREB); (**B**,**C**) Quantification of band intensities in (**A**). Data are expressed as mean ± SEM, * *p* < 0.05, ** *p* < 0.01, *** *p* < 0.001 vs. the control group.

Spatial learning and memory are dependent on the functions of the hippocampus and prefrontal cortex. Hence, we next investigated the effects of long-term administration of *Lactobacillus* strains on neuronal plasticity biomarkers, such as BDNF and CREB in the hippocampus and cortex. As shown in Figure 5A, L. K71-fed mice had significantly higher *Bdnf* mRNA levels in the hippocampus than the control group, whereas no significant increase was observed in the cortex. A similar increase was observed in BDNF protein expression (Figure 5B,C). Furthermore, L. K71-fed mice had higher BDNF staining within the dentate gyrus and CA3 region of the hippocampus compared to other groups (Figure 6). The expression of CREB and its activated form (pCREB) was also significantly upregulated in the hippocampus, but not in the cortex, of SAMP8 mice fed L. K71 (Figure 7A–C). These results suggest that long-term supplementation of L. K71 can upregulate BDNF expression by activation of the transcription factor CREB in the hippocampus of SAMP8 mice.

## 4. Discussion

The present study demonstrated that long-term administration of a diet supplemented with *Lactobacillus paracasei* K71, isolated from sake lees, could prevent age-related cognitive decline through upregulation of BDNF expression and serotonin levels in SAMP8 mice. Neurotrophic factors play key roles in neuronal development, differentiation, synaptogenesis, and survival in the brain [12]. BDNF belongs to the neurotrophin family, which plays important roles in neuronal growth, survival, and plasticity, the latter being essential for cognitive processes. During development, BDNF acts as a signal for normal axonal growth [13] and is needed for the maturation and survival of different neuronal phenotypes [14]. BDNF is also involved in synaptic plasticity [15] and is crucial for cognitive processes [16]. Age-dependent cognitive decline is characterized by perturbations in neurotransmitter synthesis and dysregulation of neurotrophic factors, particularly BDNF [17]. Therefore, sustained BDNF expression may be vital in preserving brain function during aging.

In the present study, a significant increase in the gene and protein expression of BDNF was observed in the brain hippocampus of L. K71-fed SAMP8 mice. Transcription of the BDNF and other neurotrophins is tightly regulated by several intracellular signaling pathways and the transcription factor, CREB [18,19]. Actually, our results suggest that the upregulation of BDNF expression by long-term consumption of a diet supplemented with L. K71 may be mediated by CREB and contribute to the preservation of neuronal plasticity and brain function. These results are in agreement with the findings of previous studies, such as those of long-term dietary supplementation with soy peptide [20] and green tea catechin [21]. As a brain neurotransmitter, serotonin is not only involved in mood and behavior control, and the pathophysiology of stress-related neurological disorders, but also regulates brain development and cognitive functions [18]. Gu et al. has reported that the serotonin level in the brain of SAMP8 is lower compared with that of SAMR1 exhibits normal aging patterns [22]. On the other hand, in this work, the serotonin levels in the serum and brain of SAMP8 were increased by feeding with L. K71. Indirect activation of norepinephrine and serotonin receptors by antidepressants can increase intracellular levels of cAMP and induce CREB phosphorylation [19]. This mechanism may underlie the upregulation of BDNF expression in L. K71-fed mice: Elevated brain serotonin levels may promote CREB phosphorylation, resulting in enhanced CREB-dependent transcription of target genes including *Bdnf*.

Approximately 95% of serotonin in the body is produced in the gastrointestinal tract, and the remaining 5% is localized in the brain [23]. Serotonin is synthesized from the essential amino acid tryptophan by 2 enzymes: Tryptophan hydroxylase converts tryptophan into 5-hydroxytryptophan, which is decarboxylated by aromatic L-amino acid decarboxylase to produce serotonin. On the other hand, monoamine oxygenases (MAOs) catalyze the degradation of monoamine neurotransmitters including serotonin, norepinephrine, dopamine, and other trace amines [24]. Both MAOA [25] and MAOB [26] isoforms increase in the brain during aging. Moreover, MAOB activity is also found to be elevated in Alzheimer’s disease patients [27]. Therefore, selective inhibitors of MAOs could be considered as a useful strategy in the development of agents for preventing age-related cognitive decline and neurodegeneration. We found decreased levels of serotonin-degrading enzymes, particularly MAOA, in the hippocampus of SAMP8 mice fed L. K71; however, no changes were observed in the gene expression of a serotonin-synthesis-related enzyme. Tissue serotonin can be rapidly metabolized by MAO, with the A isoform having much greater affinity for the substrate compared to the B isoform [28]. Taken together, these findings show that L. K71 administration may suppress serotonin degradation without affecting serotonin synthesis in the brain.

The 5-HT6 receptor is one of many serotonin receptors expressed in the hippocampus and associated with various cognitive processes. Preclinical studies of the 5-HT6 receptor revealed a role in the regulation of learning and memory [29]. However, no significant changes were found in the 5-HT6 receptor protein expression among the mouse groups in our experiments (Figure S2), suggesting that other serotonin receptors or combinations thereof are required for the regulation of BDNF expression. Further study will be needed to identify the specific pathways involved in BDNF expression control following the enhancement of serotonin levels in the hippocampus.

Previously, the indigenous microbiota has been reported to modulate the hippocampal levels of serotonin, indicating a role in regulating the brain serotonergic system [30]. Similarly, other studies have also shown the serum concentrations of serotonin to be significantly reduced in germ-free mice raised in the absence of microbial colonization, compared to specific pathogen-free or conventionally-colonized mice [31,32]. We thus assume that the metabolites produced by L. K71 could provide beneficial effects such as increased serotonin level in the brain and serum. Recently, Yano et al. [33] reported that indigenous spore-forming bacteria from the mouse and human gut microbiota promoted serotonin biosynthesis in colonic enterochromaffin cells and modulated serotonin concentrations in both the colon and blood. They demonstrated that specific microbial metabolites were elevated by the spore-forming bacteria, signaling enterochromaffin cells to increase serotonin synthesis. Musumeci et al. [17] also showed that chronic administration of a high-tryptophan diet increased the brain serotonin level and prevented the reduction of BDNF protein expression in the aged rat hippocampus and frontal cortex. This finding suggest that specific microbiota can promote systemic serotonin production in the gut by enhancing the availability of tryptophan. Further studies will be necessary to reveal the underlying mechanisms of the increase in serotonin metabolism and the induction of neurotrophic factor expression in the brain.

## 5. Conclusions

In the present study, we demonstrated that long-term administration of a diet supplemented with *Lactobacillus paracasei* K71, isolated from sake lees, prevents age-related cognitive decline in the SAMP8 mouse model. Our findings suggest that prolonged consumption of L. K71 may enhance serotonin levels and induce BDNF expression in the hippocampus, contributing to sustained neuronal plasticity. Daily intake of *Lactobacillus paracasei* K71 may be a promising preventative strategy for age-related cognitive decline in the elderly.

## Figures and Tables

**Table 1:** Sequences of the primers used in qPCR.

Gene	Forward Primer (5′–3′)	Reverse Primer (5′–3′)
*Bdnf*	TAATGCAGCATGATGGGAAA	ACACTGAGGCCACAATCATGC
*Tph2*	GAGCAGGGTTACTTTCGTCCATC	AAGCAGGTCGTCTTTGGGTCA
*Maoa*	GAGGCTCCAATTTCAATCACTCTG	ATGTAGTTTAGCAAGTCGTTCAGC
*Maob*	AAGCGATGTGATCGTGGTGG	CAATGAGCCAAGTGAGCGAGA
*Actb*	AGTGTGACGTTGACATCCGT	TGCTAGGAGCCAGAGCAGTA

## Supplementary Materials

The following are available online at http://www.mdpi.com/2072-6643/10/6/762/s1, Figure S1: Changes in body weight (A) and food intake (B) in mice, Figure S2: Effects of 43-week *Lactobacillus* strain administration on the expression levels of 5-HT6 receptor protein in the hippocampus and cortex of SAMP8.
