# Supplementary material for: Long-Term Diet Supplementation with Lactobacillus paracasei K71 Prevents Age-Related Cognitive Decline in Senescence-Accelerated Mouse Prone 8
Source: Nutrients. 2018 Jun 13;10(6):762. doi: 10.3390/nu10060762 (PMC6024584; doi:10.3390/nu10060762)
Supplement: Supplementary file 1 [file nutrients-10-00762-s001.pdf]

Supporting Information

Long-Term Diet Supplementation with Lactobacillus paracasei K71 Prevents Age-Related Cognitive Decline in Senescence-Accelerated Mouse Prone 8

Henry Marzo Corpuz, Saki Ichikawa, Misa Arimura, Toshihiro Mihara,
Takehisa Kumagai, Takakazu Mitani, Soichiro Nakamura and Shigeru Katayama *

*Shigeru Katayama, PhD
skata@shinshu-u.ac.jp; Tel.: +81-265-77-1603

A

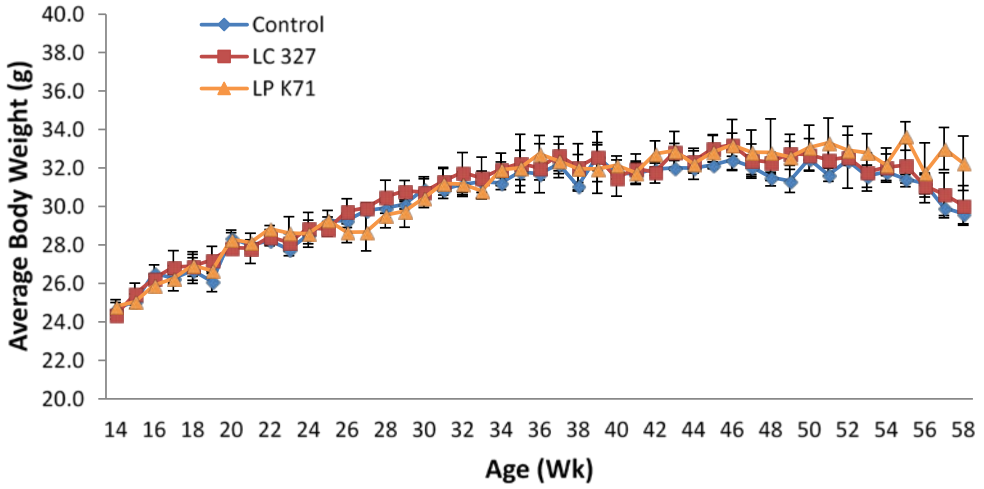

B

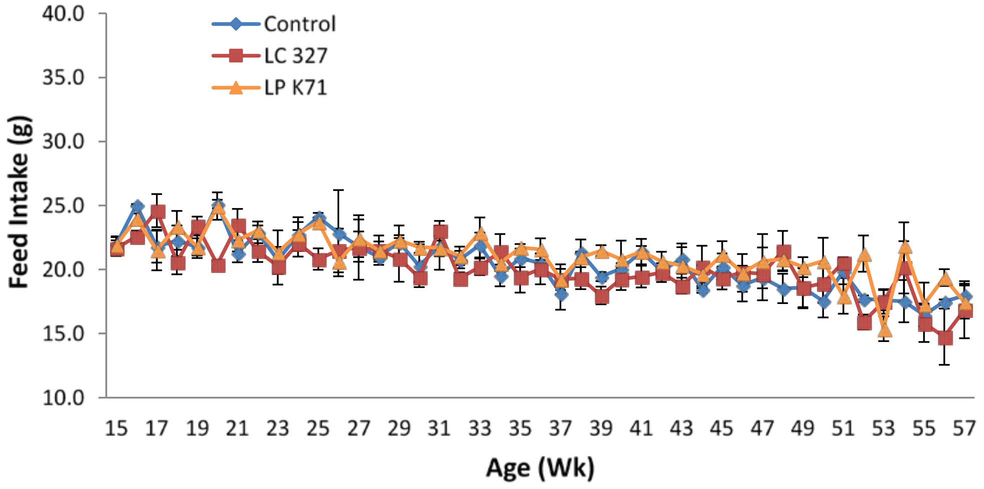

Figure S1. Changes in body weight (**A**) and feed intake (**B**) in mice. Data are expressed as mean ± SEM, n = 12/group.

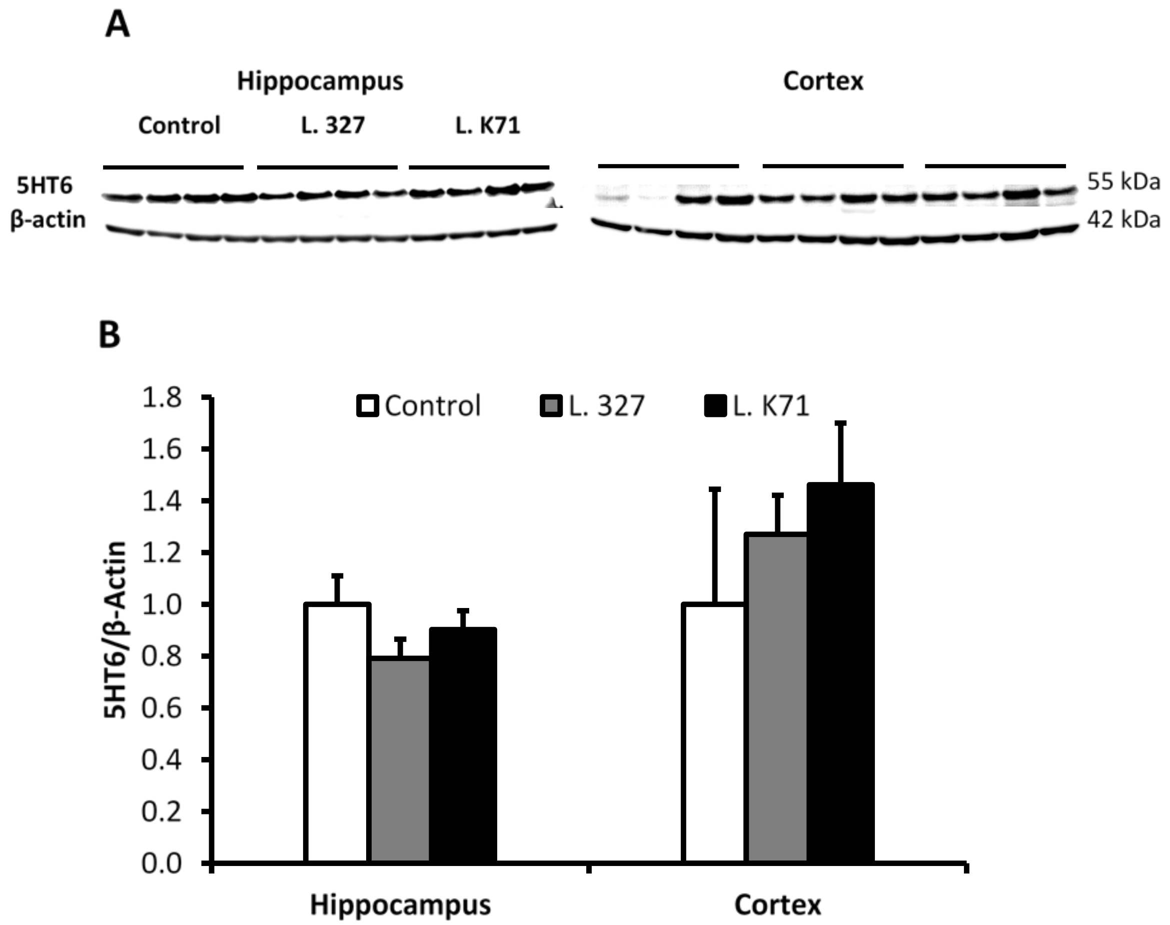

Figure S2. Effects of 43-week *Lactobacillus* strain administration on the expression levels of 5-HT6 receptor protein in the hippocampus and cortex of SAMP8. (**A**) Western blotting analysis (n = 4 mice per group) of the protein levels of 5-HT6 receptor; (**B**) Quantification of band intensities in (A). Data are expressed as mean ± SEM.
